# Assessing the Risk of Interfacility Transport in Pregnant Patients Due to Progression of Labor: Lessons From a Specialized Maternal-Fetal Transport Program

**DOI:** 10.7759/cureus.70542

**Published:** 2024-09-30

**Authors:** Thomas Lardaro, Adhitya Balaji, David Yang, Diane Kuhn, Nancy Glober, Christine M Brent, Katherine Couturier, Amelia Breyre, Julia Vaizer, Benton R Hunter

**Affiliations:** 1 Emergency Medicine, Yale School of Medicine, New Haven, USA; 2 Emergency Medicine, Indiana University School of Medicine, Indianapolis, USA; 3 Emergency Medicine, University of Michigan Medical School, Ann Arbor, USA

**Keywords:** ems, emtala, emergency medicine, pregnancy, obstetric transport, labor, interfacility transport

## Abstract

Background

Pregnant laboring patients sometimes require interfacility transfer to a higher level of care. There is a paucity of evidence to inform when it is safe to transfer a laboring patient and when delivery may be too imminent to transfer.

Methods

This is a retrospective study of pregnant patients undergoing interfacility transfer with a specialized obstetric transport team deployed from a large Midwest regional healthcare system. The primary outcome was delivery prior to or within one hour of arrival at the receiving institution due to progression of labor. Data collected included basic demographics, vital signs, gravidity, parity, gestational age, contraction frequency if contractions were present, and cervical dilation. We sought to define the association between these variables and the primary outcome to inform risk assessment for precipitous delivery among patients being considered for interfacility transfer.

Results

Of the 370 pregnant patients for whom the specialized transfer team was requested, 11 (3%) met the primary outcome. Those with more advanced cervical dilation and those who did not receive regular prenatal care were more likely to meet the criteria for the primary outcome. For every centimeter of cervical dilation, the odds of meeting the primary outcome increased 2.3-fold (95% CI: 1.5-3.4).

Conclusions

We identified risk factors for early delivery among pregnant patients for whom an interfacility transfer was requested and described patients who were high-risk for obstetric interfacility transport due to the progression of labor. Our results can help inform risk assessments for transferring potentially high-risk laboring patients.

## Introduction

Pregnant women in labor often present to hospitals that are ill-equipped to handle obstetric or newborn patients. In these scenarios, clinicians must weigh the risks and benefits of whether, when, and how to transfer the patient to a more appropriate hospital or level of care.

Given the myriad factors involved, establishing a set of standards for the appropriateness of interfacility transport for laboring patients is challenging. The incidence of precipitous delivery during transport is low, especially after the 1986 implementation of the Emergency Medical Treatment and Active Labor Act (EMTALA) in the United States [1-3]. Screening patients in labor just before transport appears to play a significant role in this regard [3]. Several studies have investigated the use of physiological signs to predict the duration of active labor [4-7]. Yet, there remains a sparsity of data on how these physiological predictors might inform the decision-making process regarding the transport of high-risk laboring patients. To our knowledge, at the time of this writing, there are no formal evidence-based algorithms published in the literature regarding this topic. Additionally, factors such as multiparity, poor antenatal care, and distance from the hospital have been associated with accidental out-of-hospital deliveries [8].

The inherent risk of a precipitous delivery during transport is of paramount concern for the interfacility transfer of high-risk patients in labor. The limited resources and physical space within which transport crews work present considerable challenges for managing the delivery and subsequent care of a mother and newborn, including any complications that may arise. Furthermore, it would be nearly impossible to secure all patients and crew members safely in the passenger compartment of a moving vehicle during any resuscitation after a delivery occurring en route. This would increase the risk of catastrophic injury to all people in the passenger compartment in the event of any type of motor vehicle or helicopter accident during such an episode. Of note, although commercial devices exist to secure healthy newborns at term during transport, large-scale safety data and validation in the literature do not exist for these products. This is also complicated by the fact that many precipitous unplanned deliveries are preterm and require neonatal resuscitation.

Lastly, there are large swaths of the United States where women have limited or no access to obstetric care or a birthing center within their county of residence. This was estimated to impact over 5 million women of childbearing age and 130,000 newborns in the United States in 2022 [9]. As a result, women in unanticipated labor in emergency situations are often far from definitive care and face longer duration transport times, thereby increasing risk.

This study aims to inform clinical decision-making regarding the timing of interfacility transport with respect to delivery by developing an evidence-based algorithm for use in risk stratifying patients being considered for transfer. Our study utilizes a unique data set from a regional high-risk maternal-fetal transport team in the Midwest to better understand what signs are associated with imminent delivery such that interfacility transport should be delayed in favor of expectant management prior to initiating transit to a receiving hospital. In order to do this, we examine patients and characteristics associated with delivery at the referring hospital or delivery within one hour at the receiving hospital due to the progression of labor.

## Materials and methods

Study design and setting

This is a retrospective case-control analysis including all pregnant patients transported for interfacility transfers by a specialized obstetric transport team deployed from the Indiana University (IU) Health system based in Indiana, from January 2021 through May 2022. The transport team consisted of a critical care transport (CCT) nurse with an expanded scope of practice through EMS protocols authorized by a physician medical director and an obstetric nurse capable of maternal-fetal monitoring, such as cardiotocography. The team is deployed by air or ground at the discretion of the sending physician (“requesting physician” in this study), with a third team member serving as a vehicle operator (pilot or driver). Team deployment is usually requested for high-risk obstetric patients, including for reasons of maternal or fetal distress not necessarily involving the progression of labor, in order to transport the patient to a higher level of care for the mother and/or neonate.

The patient encounters studied are all interfacility transports requested by a physician at a referring facility, who is often speaking to another physician, typically an obstetrician or emergency physician, at a tertiary care center who accepts the patient. By convention, the determination to deploy the specialty transport team is at the discretion of the referring physician, however, this can be influenced during a conference call with the physicians accepting the patient at the tertiary care center.

Inclusion criteria

Patient inclusion occurred through matching a database of mandatory post-transport maternal-fetal team debrief forms filed by the maternal-fetal transport teams after every patient encounter (see below) with electronic dispatch data that recorded the specialty team’s dispatch and deployment throughout the study period. The datasets were matched, linked to their patient care report in the electronic medical record (EMR) of the interfacility transport team, and reviewed as part of a quality assurance project. Any incidences of the team being dispatched without actual patient contact were excluded. Such occurrences happened only when the request for the transport team was canceled by the requesting physician prior to the team's arrival at the patient and after the team was deployed by dispatch. Over the study period, there were 12 such instances where the transport request was canceled after dispatch and prior to patient contact by the transport team.

Data acquisition

The following data elements of the EMR were collected: demographics, vital signs, gestational age, maternal heart rate, gravidity, parity, the obstetric condition requiring specialized transport, transport distance, fetal status based on fetal heart rate (FHR) and uterine contraction monitoring, cervical dilation, frequency of contractions, prenatal care status, and general notes from the patient narrative. This was collected by two authors trained in chart review and data extraction using standardized data extraction forms (Lardaro T, Balaji A). Adjudication of any uncertainty was performed by a separate board-certified emergency medicine attending physician (Hunter BR).

After all transports, the mandatory post-transport debrief form was filed by the obstetric transport nurse and included whether or not the patient was deemed too unstable to transport at the sending hospital and whether or not there was a delivery at the receiving hospital within one hour of arrival.

For the purposes of analysis, the most advanced cervical dilation and the most frequent rate of contractions documented prior to the initiation of transport were utilized.

Regular prenatal care was scored as charted by the obstetric transport nurse in the EMR - this did not have formally defined parameters and was subjectively determined by the clinician caring for the patient. In this system, the maternal-fetal nurse had three options for prenatal care documentation in the EMR: none, scant, and regular. The maternal-fetal nurses chose one category based on the history obtained from the patient using their professional discretion. CCT skill utilization was defined as any management of life-support devices beyond typical advanced life support capabilities, such as mechanical ventilation, use of vasopressors, or management of a thrombolytic drug for an acute thromboembolic event.

Table 1 summarizes the data elements and their respective methods of collection.

**Table 1:** Data elements from the EMR and their collection methods. CCT: Critical care transport.

Description	Data collection method
Maternal age	Retrieved through automated data pull from EMR
Heart rate	Retrieved through automated data pull from EMR
Blood pressure	Retrieved through automated data pull from EMR
Oxygen saturation	Retrieved through automated data pull from EMR
Respiratory rate	Retrieved through automated data pull from EMR
Gestational age	Retrieved through chart review
Gravidity	Retrieved through chart review
Parity	Retrieved through chart review
Reason for transport team request	Retrieved through chart review
Cervical dilation	Retrieved through chart review
Frequency of contractions	Retrieved through chart review
Prenatal care status	Retrieved through automated data pull from EMR
Notes from patient narrative	Retrieved through chart review
CCT skill utilization	Determined through chart review
Delivery at referring facility or within one-hour of arrival	Retrieved through chart review

Definition of primary outcome

The primary outcome was delivery prior to or within 1 hour of arrival at the receiving institution due to the progression of labor. Patients who were too unstable to transport or delivered within one hour at the receiving facility due to only maternal or fetal distress, not progression of labor, prompting cesarean delivery were not counted as having the primary outcome given that our goal was to identify patients at risk of precipitous delivery due to progression of labor. Therefore, the primary outcome did not include patients who needed delivery due to maternal or fetal emergency care situations such as maternal cardiopulmonary instability or persistent fetal bradycardia in the absence of labor.

Analysis

The OR of high-risk transports due to progression of labor in relation to various factors was estimated using simple logistic regression. Multivariate logistic analysis was not used due to the low frequency of the primary outcome (n = 11). Observations with missing values in the independent variable were dropped from the analysis. All statistical analyses were performed using Stata 18.0 BE (College Station, Texas).

## Results

Within the entire cohort, the median gestational age was 30 weeks and the interquartile range of maternal ages was 23 to 32 years, with a median of 28. A greater proportion of patients without the primary outcome received regular prenatal care. Patients with the primary outcome tended to have more advanced cervical dilation and longer transport mileage. Less than 3% of the entire cohort required CCT team skills during the encounter (Table 2).

**Table 2:** Patient and transport characteristics by outcome. *IQR: Interquartile range; CCT: Critical care transport.

Patient or Transport Characteristics	No outcome n = 359	Outcome present n = 11
Age in years, median (IQR)	28 (23-32)	28 (23-33)
Gestational age in days, median (IQR)	214 (188-233)	203 (178-224)
Parity, median (IQR)	1 (0-2)	2 (1-2)
Regular prenatal care, number (%)	311 (87)	7 (64)
Cervical dilation in centimeters (cm), median (IQR)	2 (1-3)	4.5 (3.5-9)
Transport distance in miles, median (IQR)	23 (9-48)	32 (18-117)
CCT team skills utilized, number (%)	9 (2)	0 (0)

Of the 370 pregnant patients for whom a transport was requested during the study period, 110 were deemed to be laboring at the time of request. Of those initially deemed laboring, 11 did not have a cervical examination and 15 did not have contraction frequency data charted, none of which had the primary outcome.

Eleven (3%) patients of the entire cohort were too unstable to transport or had a delivery within an hour of arrival at the receiving hospital due to the progression of labor. Of those not classified as having the primary outcome due to maternal decompensation or fetal distress, three nonlaboring patients experienced cesarean deliveries within an hour of arrival at the receiving hospital due to maternal cardiopulmonary decompensation secondary to COVID-19 pneumonia, while another three non-laboring patients had a cesarean delivery at the sending hospital due to fetal distress.

In patients with the primary outcome, the reason for the specialized transport team request was most commonly due to preterm labor or preterm premature rupture of membranes (PPROM). Most had contractions every 5 minutes or more frequently. Additionally, these patients tended to have more advanced cervical dilation. One patient had a foot presentation at the entrance of the cervix, prompting delivery at the sending hospital (Table 3). The odds of having the outcome increased 2.3 times (95% CI: 1.5-3.4) for every additional centimeter of cervical dilation (Table 4).

**Table 3:** High-risk interfacility transport requests due to progression of labor. PPROM: Preterm premature rupture of membranes.

Age (years)	Parity	Gestation (weeks, days)	Cervical dilation (cm)	Contraction frequency (minutes)	Regular prenatal care	Reason for transport request, comments, and disposition
19	1	34w4d	5.5	2	No	PPROM, cervical change more than 1 cm per hour, delivered at sending hospital
28	1	25w3d	4	3	Yes	Preterm labor, delivery within one hour at receiving hospital
29	3	22w	3	-	No	PPROM, foot presentation at entrance of cervix, delivered at sending hospital
33	2	28w	10	1	No	PPROM, delivered at sending hospital within 20 minutes of team arrival
27	0	32w	6.5	4	Yes	Preterm labor, delivered at sending hospital
32	2	29w6d	3	2	Yes	Preterm labor and diabetic ketoacidosis, regular strong contractions with urge to push, delivered at sending hospital
36	2	31w1d	3.5	1.5	Yes	Preterm labor, fetal bradycardia with immediate cesarean delivery at the receiving hospital upon arrival
24	1	34w1d	4.5	2	Yes	PPROM, regular strong contractions; cervical change over 1.5cm per hour, delivered at sending hospital
23	2	23w4d	4	5	Yes	PPROM, strong regular contractions, delivered at sending hospital
21	0	28w1d	9	2	Yes	Preterm labor, 3 cm cervical dilation in less than an hour, regular strong contractions, delivered at sending hospital
33	3	Unknown	10	2	No	Preterm labor and preeclampsia, regular strong contractions, cervix dilating more than 2cm per hour, delivered at sending hospital

**Table 4:** Simple logistic regression of various factors on the outcome of high-risk interfacility transport due to progression of labor. * Does not include patients not transported. ** Each analysis does not include observations with missing data in the independent variable.

Independent variable	Number of observations	OR	95% CI
Regular prenatal care	370	0.27	0.08-0.96
Transport distance (miles)*	352	1.01	0.99-1.03
Maternal age	359	0.99	0.90-1.10
Gestational age (days)	368	0.99	0.97-1.01
Parity	370	1.17	0.79-1.74
Cervical dilation (centimeters)	139	2.27	1.50-3.44
Contraction frequency (minutes)	130	0.96	0.71-1.30

## Discussion

**Figure 1:**
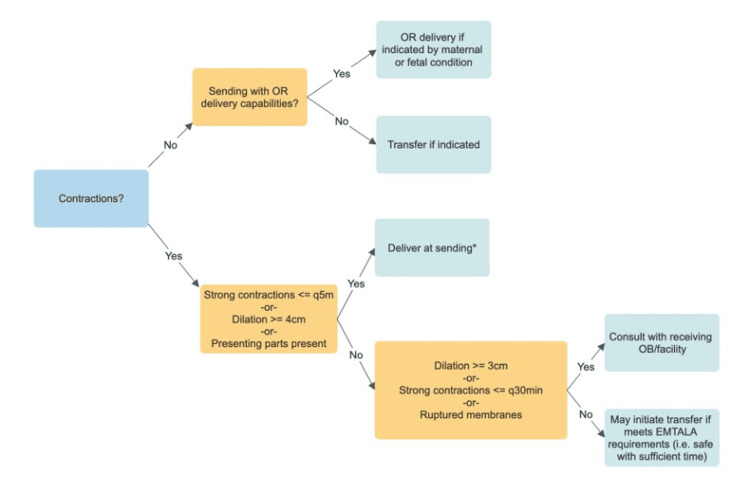
Triage algorithm for interfacility transport of pregnant patients.

Our findings add context and perspective to the clinical decision-making involved in determining whether or not a pregnant patient is safe for transport between two hospitals. The findings are amenable to formulating triage algorithms, such as Figure 1, whereby clinical teams can rapidly assess the best course of action based on the evidence presented. In particular, the findings demonstrate that preterm labor can progress rapidly, and high-risk features for precipitous delivery include the rupture of membranes, cervical dilation beyond 3 cm, and frequent contractions that are regular and strong.

It is important to note that unanticipated labor or delivery in the patient population requiring interfacility transport is largely different from the controlled progression of labor in a modern hospital labor and delivery unit. The effects of anesthesia and perinatal interventions common in labor and delivery units augment cervical change such that it is less precipitous than would be expected without any such interventions in an unplanned and potentially precipitous delivery encountered in an emergency [10,11]. Furthermore, precipitous deliveries, defined as those occurring in less than three hours, have a higher likelihood of happening outside of the controlled environment of a labor and delivery unit [11]. As such, findings from in-hospital labor and delivery units are not necessarily representative of the high-risk and unanticipated deliveries encountered in the prehospital and interfacility transport environments. Our findings help fill in this gap and provide better context for clinical decision-making as it relates to the interfacility transport of high-risk obstetric patients.

Another clinical context in which these findings are useful is for clinicians balancing the risk of adverse events during transport, the requirements of EMTALA in the United States, and the potential benefit of a neonate requiring higher-level care being born at an appropriately resourced hospital capable of delivering that care. In this study, nearly all the neonates born in the group with the primary outcome would benefit from advanced neonatology capabilities, as all had gestational ages less than 35 weeks, and the majority were less than 30 weeks.

As healthcare resources within the United States continue to consolidate and centralize, accessing prenatal care and obstetric services will remain a challenge. In this dataset, we found an association between high-risk deliveries and the absence of prenatal care. Lack of access to care in obstetric patients prevents medical optimization and planning for high-risk pregnancies. Without improved access, adverse events such as unexpected preterm deliveries could become more frequent with higher morbidity and mortality. Furthermore, patients with difficulty accessing any type of healthcare could also conceivably present later in the course of an acute health crisis, further decreasing the odds of a favorable outcome.

Lastly, the results imply that with adequate fetal, uterine, and maternal clinical assessment capabilities, there is limited utility in an obstetric transport nurse program if an appropriate triage mechanism is in place. Furthermore, the deployment of the obstetric transport nurse led to a mean increase in response time to the patient of over twenty minutes compared to a standard transport team configuration (data not shown). This was due to the standard transport team having to divert to pick up the obstetric nurse from a centralized labor and delivery unit that was away from the point of ambulance dispatch. In the era of staffing shortages exacerbating a lack of adequate nursing coverage within hospitals in the United States [12-14], these findings may inform more efficient deployment of valuable and limited nursing resources.

Limitations

This study had several limitations worth considering. First, there was implicit selection bias in that the obstetric transport team configuration was deployed at the behest of the requesting physician at the sending hospital for pregnant patients presumably perceived to be high risk. The patient population therefore contained a preponderance of preterm patients with more comorbidities than would be expected in a more generalized sample. While it is possible that transports occurred by other means, it is highly unlikely in this referral area because this was the only service with this capability operating regionally. Transports by other means would have had to utilize local 9-1-1 resources to travel long distances outside of normal response areas, leaving the community for long periods of time.

A second source of potential bias comes from the physicians involved in arranging acceptance for transfer and transport. The outcome was rare and could be subjectively influenced by the clinical decision-making of clinicians at both the sending and receiving hospitals. The transport team has real-time access via telephone to an online medical control obstetric physician for any questions or concerns. The online medical control physician can place orders and has the authority to decline immediate transport in favor of delivery at the sending hospital prior to transport. Reasons for postponing transport may include imminent delivery and/or fetal or maternal distress, such as eclampsia or prolonged recurrent decelerations on fetal heart tracing, or if the transport team or online medical control physician feels that delivery at the sending hospital is possible and indicated prior to transport. Practice bias in either group will lead to non-random findings as it relates to the outcome and physiological parameters. For example, the presence of prolonged, recurrent late decelerations often led to cesarean delivery if that capability was present. The same is likely true of varying degrees of cervical dilation and the nature of contractions.

Another potential limitation is the speed at which patients who stayed at the sending hospital were delivered. While many of the cases were taken directly to the OR, there is some uncertainty in how precipitous the labor was, although the rate of cervical change in all but three of the cases implies precipitous delivery. Missing data existed in this dataset for patients who did not have a cervical exam performed or who did not have contraction frequency measured. These observations were not included in the logistic analysis. This diluted the power of the study and the ability to precisely measure the impact of these factors. Lastly, the study took place within a single region of the United States with the least favorable quartile of morbidity and mortality for both maternal and neonatal patients in the perinatal period. Thus, the patient population described may have worse outcomes than would be expected in other regions with better healthcare resources and/or availability thereof.

## Conclusions

The findings of this study imply that patients with regular strong contractions, preterm labor, cervical dilation beyond 3 cm, or rupture of membranes are at high risk for progression of labor and precipitous delivery during interfacility transfer. The findings suggest that real-time obstetric physician consultation with up-to-date clinical context, such as cervical dilation, uterine contraction frequency, and FHR, can help mitigate risk prior to transfer at the sending hospital. Developing a local algorithm that supports physician judgment with the incorporation of clinical factors and distance to the receiving hospital in the decision of whether or not to undertake these high-risk transfers can help mitigate risk in the transfer of obstetric patients experiencing labor.
